# Observations on the Cause and Cure of the Tetanus

**Published:** 1786

**Authors:** Benjamin Rush

**Affiliations:** Professor of Chemistry in the University of Pensylvania


IX. *Obſervations on the Cauſe and Cure of the Tetanus. By* Benjamin Ruſh, *M. D. Profeſſor of Chemiſtry in the Univerſity of Penſylvania.*— From the *Tranſactions of the American Philoſophical Society, held at Philadelphia, for promoting uſeful Knowledge.* Vol. II. 4to. Philadelphia, 1786.

DURING my attendance upon the military hoſpitals of the United States, in the courſe of the late war, I met with ſeveral caſes of the tetanus. I had frequently met with this diſorder in private practice, and am ſorry to ſay that I never ſucceeded with the ordinary remedy of opium in any one caſe that came under my care. I found it equally ineffectual in the army. Baffled in my expectations from a remedy that had been ſo much celebrated, I began to inveſtigate more particularly the nature of the diſorder. I found it to be a diſorder of warm climates, and warm ſeaſons. This led me to aſcribe it to relaxation. I reſolved to attempt the cure of it by a ſet of medicines in ſome meaſure the oppoſites of moſt of the medicines that had been employed in that diſorder. Soon after I adopted this reſolution, I was called to viſit Colonel John Stone, who who was wounded through the foot at the battle of Germantown on the 4th of October 1777. He was in the third day of a tetanus. His spasms were violent and his pains so exquisite that his cries were heard near a hundred yards from his quarters. His head was thrown a little backwards, and his jaw had become stiff and contracted. He was under the care of a skilful regimental surgeon who was pouring down opium in large quantities without effect.

Duty and friendship both led me to do my utmost to save the life of this valuable officer. I immediately dismissed the opium, and gave him large quantities of wine and bark, to the amount of two or three ounces of the latter, and from a bottle to three pints of the former in the day. In a few hours I was delighted with their effects. His spasms and pains were less frequent and violent, and he slept for several hours, which he had not done for several days and nights before.

With the same indication in view, I applied a blister between his shoulders, and rubbed in two or three ounces of mercurial ointment upon the outside of his throat. He continued to mend gradually under the operation of these medicines, so that in ten days he was out of

 danger, danger, although the ſpaſm continued in his wounded foot for ſeveral weeks afterwards. In the ſummer of the year 1782 I was called to viſit a ſervant girl of Mr. Alexander Todd, merchant of this city, who had brought on a tetanus by ſleeping in the evening on a damp brick pavement, after a day in which the mercury in Farenheit's thermometer had ſtood at near 90°. The caſe was nearly as violent and alarming as the one I have deſcribed. I treated her in the ſame manner, and with the ſame ſucceſs. To the above-named medicines, I added only the oil of amber which ſhe took in large doſes, after I ſuſpected the tonic powers of the bark and wine began to loſe their effects. The good effects of the oil were very obvious. She recovered gradually and has continued ever ſince in good health. In the ſummer of the ſame year I was called to Alexander Leſlie, a joiner, who had run a nail in his foot. I found him the day afterwards in extreme pain, with ſmall convulſions and now and then a twinge in his jaw. The wound in his foot was without ſwelling or inflammation. I dilated the wound and filled it with lint moiſtened with ſpirit of turpentine. This in a little while produced a good deal of pain and a great inflammation in his his foot. While I was preparing to treat him in the manner I had treated the two former cases, the pains and spasms in his body suddenly left him, and in twenty-four hours after I saw him, he complained of nothing but of the pain and swelling in his foot, which continued for several weeks and did not leave him till it ended in a suppuration. From the history of these three cases, I beg leave to make the following remarks.

1. That the predisposition to the tetanus depends upon relaxation. This relaxation is generally produced by heat; but excessive labour, watchings, marches, or fatigue from any cause, all produce it likewise, and hence we find it more frequent from wounds received in battles, than from similar wounds received in any other way. These wounds more certainly produce the tetanus, if they have been preceded for some time with warm weather. Dr. Shoepft, the physician general of the Anspach troops that served at the siege of York in the year 1781, informed me of a singular fact upon this subject. Upon conversing with the French surgeons after the capitulation, he was informed by them that the troops who arrived just before the siege from the West Indies with Count de

 Grasse,

Graſſe, were the only troops belonging to their nation that ſuffered from the tetanus. There was not a ſingle inſtance of that diſorder among the troops who had ſpent a winter in Rhode Iſland.

2. As the tetanus ſeems to be occaſioned by relaxation, the medicines indicated to cure it are ſuch only as are calculated to remove this relaxation and to reſtore a tone to the ſyſtem. The bark and wine appear to act in this way. The operation of the bliſters, is of a more complicated nature. That they are ſedative and antiſpaſmodic in fevers is univerſally acknowledged, but in the peculiar ſtate of irritability which occurs in the tetanus, perhaps their effects are more ſimply ſtimulating. But I will go one ſtep farther. In order to cure this diſorder, it is neceſſary not only to produce an ordinary tone in the ſyſtem, but ſomething like the inflammatory diatheſis. The abſence of this diatheſis is taken notice of by all authors, particularly by Dr. Cullen *.

Mercury appears to act only by promoting this diatheſis. Hence it never does any ſervice unleſs it be given time enough to produce a

* Firſt Lines, Vol. III.

ſalivation, falivation. The irritation and inflammation produced in the mouth and throat, feldom fail to produce the inflammatory diathefis, as blood drawn in a falivation has repeatedly fhewn.

I apprehend that the oil of amber acts as a ftimulant chiefly in this diforder. I have heard of a tetanus being cured in the ifland of Grenada by large dofes of muftard. Dr. Wright, lately of the ifland of Jamaica, relates in the 6th volume of the London Medical Effays, feveral remarkable cafes of the tetanus being cured by the cold bath. Both thefe remedies certainly act as ftimulants and tonics. By reafoning, *à priori*, I conceive that electricity would be found to be an equally powerful remedy in this diforder.

As a general inflammatory diathefis difpofes to topical inflammation, fo topical inflammation difpofes to general inflammatory diathefis. Wounds, upon this account, are lefs apt to inflame in fummer than in winter. In the tetanus I have uniformly obferved an abfence of all inflammation in the wounds or injuries that produced it. A fplinter under the nail produces no convulfions, if pain, inflammation and fuppuration follow the accident. It is by exciting pain and inflammation I apprehend that the fpirit of turpentine acts in all wounds and punctures of nervous and tendinous parts. I have never known a ſingle inſtance of a tetanus from a wound, where this remedy had been applied in time. It was to excite an inflammation in the foot of Mr. Leſlie, that I dilated the wound and filled it with the ſpirit of turpentine. I was not ſurpriſed at its good effects in this caſe, for I was prepared to expect them.

I find a remarkable caſe related in Dr. W. Munro's Theſis, publiſhed in Edinburgh in the year 1783, of a black girl who had a tetanus from running a nail in her foot, being perfectly cured by deep and extenſive inciſions being made in the wounded part by Dr. John Bell, of the iſland of Grenada.

It is by producing inflammation in a particular part, and tone in the whole ſyſtem, I apprehend that the amputation of a wounded limb ſometimes cures a tetanus; and it is becauſe the degrees of both are too inconſiderable to oppoſe the violence of the ſpaſms in the advanced ſtages of the tetanus, that amputation often fails of ſucceſs.

I have been informed by a phyſician who reſided ſome time at St. Croix, that the negroes on that iſland always apply a plaſter made of equal equal parts of ſalt and tallow to their freſh wounds, in order to prevent a locked jaw. The ſalt always produces ſome degree of inflammation.

If the facts that have been ſtated are true, and the inferences that have been drawn from them are juſt, how ſhall we account for the action of opium in curing this diſorder? I do not deny its good effects in many caſes, but I believe it has failed in four caſes out of five in the hands of moſt practitioners. It is remarkable that it ſucceeds only where it is given in very large doſes. In theſe caſes I would ſuppoſe that its ſedative powers are loſt in its ſtimulating. It is upon a footing, therefore, in one reſpect, with the ſtimulating medicines that have been mentioned; but from its being combined with a ſedative quality, it is probably inferior to moſt of them. I am the more inclined to adopt this opinion, from an account I once received from Dr. Robert, of the iſland of Dominique, who informed me that after having cured a negro man of a tetanus with large doſes of opium, he was afterwards ſeized with a diſorder in his ſtomach, of which he died in a few days. Upon opening him, he found his ſtomach inflamed and mortified. I

do not forbid the ufe of opium altogether in this diforder. I think fmall dofes of it may be given to eafe pain, as in other fpafmodic diforders; but as its qualities are complicated, and its efficacy doubtful, I think it ought to yield to more fimple and more powerful remedies.

To the cafes that have been mentioned, I could add many others, in which I have reafon to believe that the excitement of a topical inflammation by artificial means, has effectually prevented a tetanus.

To this account of the tetanus, I beg leave to fubjoin a few words upon a diforder commonly called the jaw-fall in infants, or the Trifmus Nafcentium of Dr. Cullen, which is nothing but a fpecies of tetanus.

I have met with three cafes of it in this city, all of which proved fatal. The ftage of the diforder in which I was confulted, and the age and weaknefs of the infants, forbade me to attempt any thing for their relief. I have introduced the fubject of this diforder in children, only for the fake of mentioning a fact communicated to me by the late Dr. Cadwalader Evans of this city. This gentleman practifed phyfic for feveral years in Jamaica, where he had frequent quent opportunities of feeing the tetanus in the black children. He found it in every cafe to be incurable. He fuppofed it to be occafioned by the retention of the meconium in the bowels: This led him invariably to purge every child that was born upon the eftates committed to his care. After he adopted this practice, he never met with a fingle inftance of the tetanus among children.

Perhaps it may tend to enlarge our ideas of the tetanus, and to promote a fpirit of inquiry and experiment, to add, that this diforder is not confined to the human fpecies. I have known feveral inftances of it in horfes from nails running in their feet, and other accidents. It is attended with a rigidity of the mufcles of the neck, a ftiffnefs in the limbs, and fuch a contraction of the jaw as to prevent their eating. It is generally fatal. In two cafes I had the pleafure of feeing the difeafe perfectly cured by applying a potential cauftic to the neck under the mane, by large dofes of oil of amber, and by plunging one of them into the river, and throwing buckets of cold water upon the other.

How far the reafonings contained in this paper may apply to the hydrophobia, I cannot determine, having had no opportunity of feeing the difeafe fince I adopted thefe principles; but from the fpafmodic nature of the diforder, from the feafon of the year in which it generally occurs, and, above all, from the cafe related by Dr. Fothergill, of a young woman having efcaped the effects of the bite of a mad cat by means of the wound being kept open, (which, from its feverity, was probably connected with fome degrees of inflammation) is it not probable that the fame remedies, which have been ufed with fuccefs in the tetanus, may be ufed with advantage in the hydrophobia? — In a difeafe fo deplorable, and hitherto fo unfuccefsfully treated, even a conjecture may lead to ufeful experiments and inquiries.

